# Observations on Pulmonary Consumption

**Published:** 1830-05

**Authors:** Joseph Parrish


### PULMONARY CONSUMPTION.

### *Observations on Pulmonary Consumption.*

By Joseph Parrish, M.D.

The following remarks on pulmonary consumption are confined to that form of the disease which is characterised by the presence of tubercles in the lungs, and which I believe to be essentially scrofulous in its nature, and very generally dependent on hereditary predisposition. I do not intend to enter into a description of the symptoms, or into any discussion of the pathology of this formidable complaint; my object is simply to present to the medical public certain practical views, which have resulted from an experience of many years, and an observation which circumstances have strongly directed into this particular channel.

I do not, however, claim for these views the credit of originality: they are similar to those which have been taken by others, and are such as may very naturally occur to any practitioner of enlarged experience. But as different opinions are entertained by men eminent in the medical profession, the testimony of one who has seen much of the complaint may not be without utility in assisting the young physician to form a sound and enlightened judgment.

At the period of my entrance into the profession, the opinion was very prevalent that consumption was an inflammatory disease, requiring in its treatment a vigorous employment of the various antiphlogistic measures. Frequent bleedings, a rigid system of diet, confinement in rooms of a regulated temperature, with the use of local depletion and of medicines calculated to lessen the force of arterial action, constituted the plan of management then fashionable both in our hospital and in the private practice of the city. It was the course pursued and recommended by that great and distinguished physician and professor, Dr. Rush, who also conjoined with it, in many instances, the liberal use of mercury. It is right, however, to state that these measures were confined to the stage of the disease which he denominated inflammatory. In the last stage, he supposed that the degree of action was below that of health, and was accustomed to allow his patients animal food with malt liquors to fill their blood-vessels, and opiates to quiet irritation. Other practitioners, less attached to the free use of the lancet and to mercury, resorted to other measures of a different, but scarcely less energetic, character. My highly esteemed and lamented preceptor, the late Dr. Wistar, was strongly attached to digitalis, which he prescribed very freely, and, in one instance which fell under my notice, with apparently very favorable results. With these active measures, others of a milder character were united, such as blisters, issues, palliatives to allay cough, &c.; but, whichever plan was adopted, the poor patients generally went one course, and that rapidly; they died.

When I began the practice of my profession, I was much disposed to pursue the course of treatment recommended by Dr. Rush, captivated, as were nearly all who attended his instructions, by his peculiarly happy method of conveying his sentiments, and of recommending them by ingenious and forcible illustrations. The experience, however, of a few years was sufficient to convince me that a change was necessary. My doubts were, indeed, very early excited by the opposite results of two apparently very similar cases of the disease, which occurred at the same time and in the same neighbourhood, and one of which I myself attended in consultation with Dr. Rush and another highly distinguished physician. I will relate these cases, as strongly illustrative of the inefficacy and injury of vigorous medical treatment in this complaint.

The patient whom I attended was a young man, not more than twenty-four years of age. As it was in the beginning of winter that we were called to the patient, our first object was to obviate the effects of the weather: we therefore had him placed in a spacious apartment, the air of which was maintained at a uniform temperature by night and day throughout the season. The treatment at first consisted in a system of rigid dieting, with small and frequent bleedings, and the use of mercury. Ptyalism, however, could not be induced; and, as the patient grew worse under the present plan, we laid aside the mercury, and resorted to diaphoretics. Sulphur and tarwater were also prescribed, under the impression that they had been useful in similar cases; but our remedies appeared to make no impression on the disease, which marched steadily forward. Various medicines were afterwards used, with little advantage: among the rest, acetate of lead, which was given in the dose of two grains every two hours for several days in succession, with the effect of diminishing the frequency of the pulse; but, as it induced symptoms of colic, we were under the necessity of abandoning it. At this period I visited the patient twice every day, and my two colleagues every morning; and nothing was omitted which occurred to the experience or sagacity of those highly distinguished physicians as likely to be productive of benefit. At length the stage of the disease arrived in which a supporting treatment was thought to be required. A stimulating diet, with tonics of various kinds, was now resorted to; but all our efforts were unavailing. The patient came under our care in the early part of winter, and died before the close of the following spring.

In the immediate neighbourhood of this young gentleman resided a student of medicine, afterwards a respectable practitioner. In the preceding summer, while resident pupil in the Pennsylvania hospital, he had been attacked with hæmoptysis, in the treatment of which a vigorous course of depletion had been pursued. The hæmoptysis disappeared, but was followed by considerable debility. A visit to the country did not restore his health, and he returned in the fall too unwell to resume his duties in the hospital. At the commencement of winter, any one, upon observing the two patients, would have supposed that the student was further advanced in the disease, more reduced, and more likely to sink than his unfortunate neighbour. He was pallid, emaciated, had cough and fever; in fact, exhibited all the marks of confirmed consumption. He resisted, however, all attempts to induce him to submit to medical advice, from a belief that the practice which would be adopted would tend only to hasten a fatal issue. The winter passed with no other treatment than the occasional use of slight palliatives, as paregoric to allay cough; and the spring which saw our patient carried to the grave, opened upon this young gentleman still alive. As soon as the weather permitted, he went into the country, and furnishing himself with a horse, sick and debilitated as he was, commenced the life of a country doctor. Strange as it may appear, he rode himself into perfect health. He acquired an extensive practice, married, and became the father of several children. He afterwards returned to the city, and fell a victim to typhous fever, contracted during his attendance upon the business of the dispensary. This happened ten or twelve years after the winter above alluded to; and not a symptom of his former complaint was observable for a long time before the period of his death. I often conversed with this gentleman relative to his case, and remember being told by him that he found no remedy so effectual in relieving his distressing chilliness as a ride on horseback. In the midst of a chill, while sitting by a large fire, having the back of his chair covered by a thick coat or blanket, and yet unable to keep himself warm, he would receive a message from a patient at a distance requiring him to mount his horse. Almost immediate relief would be experienced from the exercise, and a ride of a few miles would produce so much excitement as to restore him to comfortable warmth.

A comparison of these two cases, so differently treated, and so different in their results, induced me very soon to doubt the propriety of that energetic practice to which I had before been inclined. Frequent opportunities afterwards occurred to me of witnessing the effects of rigid antiphlogistic treatment; and their almost invariably unfavorable character strengthened the suspicion of the inefficiency, and even injury, of such a course. I have repeatedly seen patients shut up in their rooms, subjected to frequent bleedings, and confined to a low diet; but I have never seen the disease yield to these measures; and a perseverance in them has appeared to me almost always to wear out and exhaust the powers of the system, and to hasten, instead of protracting, the period of dissolution. After a long course of observation and experience, having witnessed the failure of every mode of management which consisted in the employment of active medicinal agents, or required frequent professional interference, I at length came to the conclusion that neither depletion, nor any medicine, nor any succession or combination of medicines, was equal to the cure, or even to the material alleviation of the complaint.

We hear, indeed, that phthisis is an inflammatory disease, and consequently requires the treatment which, by universal consent, is applied to other affections of this character. Rest, low diet, and depletion, will subdue inflammation: why should they not be adequate to the cure of consumption? Theory points them out as appropriate remedies; experience is a fallacious guide; obey, therefore, the dictates of reason, and if the case should terminate unfortunately, your patient will at least die *secundum artem*, and you will have this consolation, that he ought to have recovered.

But I do not believe that the condition of the lungs in tuberculous phthisis is at all analogous to ordinary inflammation, nor that it is to be treated upon similar principles. The diseased action is peculiar, and requires peculiar modes of cure. It is true that genuine inflammation may, and often does, supervene upon the proper phthisical complaint, and tends to aggravate it. It is no uncommon circumstance for consumptive patients to be attacked with catarrh, and with acute pain in some part of the thorax. In this case, moderate depletion, either general or local, together with blisters to the breast, and other antiphlogistic measures, may be employed with advantage; but they should even here be used with caution, for fear of producing a degree of debility which the system, in its diseased condition, may not be able to surmount. But, merely with the view of curing the tuberculous affection, bleeding should never be used: without diminishing the disordered action, it tends to enfeeble those vital energies which constitute the only defence against the progress of the invader.

The pulse, it is said, is strong, accelerated, and requires to be reduced; but it is a pulse of irritation, not of inflammation. Such a pulse you will often find, in hectic patients, continue to the very latest period of life, while all the other actions of the system are sunk in the lowest debility. Such a pulse I have known to be still more excited by the repeated use of the lancet; and it is often reduced and brought down to the natural standard by a contrary plan of treatment. I recollect a case of pulmonary consumption, in which a rigid antiphlogistic diet, adhered to for some time, had of itself the effect of producing a great increase in the excitement of the pulse, which was allayed by a change to more nourishing food. The circulation even of a healthy individual may be brought into an irritated state by depriving the system of that support from a due degree of food, air, and exercise, which is essential to the preservation of a just balance in all its operations. Take a robust man, confine him in a close room, bleed him repeatedly, diet him strictly, keep up action in his bowels by purgative medicine, and allow not a breath of air to blow upon him, and, if I am not greatly mistaken, his pulse will become frequent and irritated, with the occurrence of night sweats, and perhaps sizy blood; in fact, that very condition of system will be produced, for which, in cases of phthisis, these measures are recommended as remedies. Now, it is well known that in consumptive patients there is generally a preternatural irritability; and it is reasonable to infer that this irritabitity must be augmented by the means which are sufficient to produce it in a healthy man; so that the system will thus be rendered less capable of resisting the operation of morbid causes, and may sink under the local disease which it might have otherwise withstood for many years, perhaps ultimately have surmounted.

But, though the antiphlogistic course of treatment, and the use of powerful medicines, are calculated rather to co-operate with the disease of the lungs in reducing the system of the consumptive patient, than to relieve or eradicate the complaint, yet we are not therefore to surrender all hope, and yield up the sufferer an unresisting victim. On the contrary, much may be done by exertion on the part of the individual affected in controlling the disease; and instances are not wanting in which it would seem to have been entirely conquered.

Vigorous exercise, and free exposure to the air, are by far the most efficient remedies in pulmonary consumption. It is not, however, that kind of exercise usually prescribed for invalids, an occasional walk or ride in pleasant weather, with strict confinement in the intervals, from which much good is to be expected. Daily and long-continued riding on horseback or in carriages over rough roads, is, perhaps, the best mode of exercise; but where this cannot be commanded, unremitting exertion of almost any kind in the open air, amounting even to labour, will be found highly beneficial. Nor should the weather be scrupulously studied. Though I would not advise a consumptive patient to expose himself recklessly to the severest inclemencies of the weather, I would nevertheless warn him against allowing the dread of taking cold to confine him on every occasion when the temperature may be low or the skies overcast.

I may be told that the patient is often too feeble to be able to bear exertion; but, except in the last stage, where every remedy must prove unavailing, I believe there are few who cannot use exercise without doors; and it sometimes happens that they who are exceedingly debilitated find, upon making the trial, that their strength is increased by the effort, and that the more they exert themselves, the better able they are to support the exertion.

It is said by those who oppose this kind of treatment, that the lungs are in a state of inflammation, and therefore require rest. But, admitting for a moment that the tubercles in pulmonary consumption are the result of ordinary inflammatory action, of a chronic or subacute character, (an opinion, however, which I have already disclaimed,) yet the argument will not hold in the present case; for, from their very organization, the lungs cannot be at rest. From the moment we begin to breathe to the latest period of life, they are necessarily in continual motion. We cannot confine them as we can a diseased joint; and in attempting to restrain their motions by keeping the body at rest, without gaining our object, we generate a degree of irritability of system, which enables the local affection to operate upon the general health with a vast increase of deleterious effect.

I was much pleased to meet with a confirmation of my views relative to the management of pulmonary consumption, in the following extract from a work of Dr. Colin Chisholm, whose experience entitles his opinion to great respect. "An active, bustling occupation of time," he says, "with exposure to what may be called and deemed hardships, such as occur in military service during an active campaign, or in maritime service of any kind, have sometimes produced a most wonderful change in a constitution broken down by phthisis. I have known instances of officers in both services recovering their health by seemingly inconsistent means. One thing is most certain, that confinement to the atmosphere of a room, or even house, is most highly prejudicial: it renders the person infinitely more susceptible of the impressions of cold, and thereby tends to augment the evil which it is supposed calculated to remedy."

By relating a few instances of the good effects resulting from the mode of life above recommended, I shall perhaps be able to give greater weight to the sentiments which I have advanced. The remainder of this paper will therefore be occupied with practical illustrations of the influence of exertion on the part of the patient, in suspending or curing the complaint.

Dr. Baldwin had a strong hereditary predisposition to pulmonary consumption. He had lost a father and several brothers with the disease, and was himself attacked with it soon after he graduated. Aware of the nature of his symptoms, and convinced that fatal consequences must result unless he should depart, in his own case, from the ordinary routine of practice, he determined to try the effects of a change of climate, united with unusual bodily exertion. In the midst of winter he embarked for Savannah in Georgia. This happened at least five or six years before his death. On his arrival at Savannah, he made up his mind to travel on foot to Milledgeville, which was distant upwards of one hundred miles. His health was at this time so much impaired, that his friends at Savannah were disposed to consider him little short of a maniac. Disregarding, however, their representations, he set off on a pedestrian journey. As the country was but thinly settled, he endured many hardships and privations, being sometimes compelled to wade through streams, and often taking up his lodging in cabins, among people as untutored as the Indian, and partaking of their homely fare of ham and corn bread. Having passed the winter at the house of a friend, he found himself, upon the opening of summer, nearly restored to health. Soon afterwards, a commission appointing him naval surgeon was received, which he was induced to accept; and, having settled in the country, he remained there for several years free from disease. On the fitting-out of the expedition to the Yellowstone river, he accepted the appointment of botanist, and, having been attacked, as I was informed, by his old complaint, died upon the journey. There can be no doubt that his disease was suspended several years by the course of life which he pursued.

A young physician, who had been a pupil of my own, was, soon after entering into practice, attacked with fistula in ano, for which I operated upon him. The fistula was small, but much indisposed to heal; and a long time elapsed before a cure was effected. Very soon afterwards he was attacked with hæmoptysis, from which he had scarcely recovered, when the symptoms of consumption became manifest. I watched the progress of the case with great solicitude. Necessity compelled him to use great exertion to gain a livelihood, and I was often surprised to find him at one time confined by an attack of spitting of blood, and a few days afterwards running about among his patients in the lanes and alleys of the city. I am convinced that his life was protracted at least one year longer than it otherwise would have been, by the great exertion to which the stimulus of necessity excited him.

Similar effects I have repeatedly witnessed in other cases. During my attendance in the Philadelphia Dispensary, my attention was occasionally attracted by patients affected with consumption, whose situation in life left them only the alternative of labouring for their support, or of becoming tenants of the Almshouse. The strong aversion entertained by many of the poor to entering the institution, serves as a powerful incentive to exertion; and I remarked that some patients, who were by this cause induced to struggle by their labour for a support to the very last, continued longer and bore their disease better than others whose circumstances were considered much more comfortable.

From a fine, healthy-looking practitioner of New England, who some years ago brought me a letter of introduction, I received the following account: In early life he had been affected with pulmonary consumption, and, while still labouring under the disease, had commenced the life of a country doctor. As there happened to be an uncommon degree of sickness in the neighbourhood at this period, he was compelled to make great and unusual exertion; and the result was a perfect restoration to health. In the course of conversation he said to me, (I copy his words,) "I have left a patient at home labouring under pulmonary consumption, with directions to ride ten miles every day, be the weather what it may." This was in the winter season.

The following notice, handed me by Dr. Gilman, was drawn up by a gentleman of Ohio, the father of the Doctor, and dated Marietta, 1823: "In the year 1804, Thaddeus M. Harris, a clergyman, gave me the following account. He had left Dorchester, Massachusetts, that spring, so low in consumption, that neither he nor any of his friends had an idea that he would be able to reach Hartford, Connecticut, distant one hundred miles. He arrived there, however; and, though still very weak, he was encouraged to prosecute his journey to New York. When there, finding that he was gaining strength, he determined to proceed to the western country. On his arrival at Marietta, he was so well as to be able to ride forty miles a day to preach, and was in fact quite recovered. He returned to his parish in Dorchester in good health; and the last time I heard from him, which was about two years since, he was still well."

A gentleman of this city, when a young man, came under my care during the winter season, affected with cough and hectic fever. I felt great solicitude in his case, and determined to try the effect of horseback exercise. In compliance with my advice, he rode daily through the winter, and in the spring was evidently improved. The summer opened upon him still affected with alarming symptoms. It was during the late war, and in the course of the season camp Dupont was formed. The young man joined one of the volunteer companies, marched down with the rest, and was subjected to all the hardships of a camp life. His health and strength increased; and he is now a hearty man, free from all signs of pulmonary disorder.

Another very interesting case occurred to me, with a similar result. A little son of a citizen of Philadelphia was affected with cough, hectic fever, profuse sweats, and great emaciation; and there was every reason to believe that his lungs were affected with tuberculous disease. Entertaining the conviction that any active medical treatment would ensure a fatal termination to the case, I strenuously opposed the use of remedies which were pressed upon the parents by the kindness of their friends. Among the rest, mercury was proposed. Happily they were disposed to listen to the suggestions of their physician, and no active treatment was employed during the winter. At the opening of spring, the child was sent into the country, with directions that he should have the benefit of free exercise and the open air. He returned free from the pulmonary complaint, has since passed through the hooping cough, and remains well to this time.

I shall close this list of cases, by giving one more illustration of the comparative effects of exercise and of confinement in the cure of consumption. Shortly before the death of Dr. Wistar, a young lady was brought from New Jersey, to consult him for chill, fever, pain in the breast, cough, and considerable loss of voice. These symptoms presented a gloomy prospect. The Doctor being absent on a journey when the lady arrived, she came under my care; and I had seen her several times before his return. We afterwards visited her together, and made a very minute examination of her case. Dr. Wistar, having been travelling over the mountains, in which mode of life he took great delight, was fully prepared to appreciate the effects of air and exercise. We had retired for the purpose of a consultation, and had re-entered the apartment, when, looking round the room, he made this remark to me: "Doctor, do you not think it looks confined and close here? Do you not think it would be best to send her back to the country, and direct her to ride every day?" I concurred heartily in the proposition. We put a seton in her side, and then advised her to return home, and use exercise. The winter passed over; the following summer she visited Philadelphia greatly improved in health, and, as I afterwards learned, became perfectly well.

About the same time with the above case, I attended another young lady, in consultation with a practitioner of the highest respectability in this city. The patient had been attacked with hæmoptysis, and was now labouring under hectic fever and cough. It was suggested in this case, that the disease was of an inflammatory character, and was to be encountered by depletion, low diet, and perfect rest. The last was considered a point of peculiar importance. The plan was put in operation. As the patient continued to grow worse, other remedies were tried; but were all of no avail. She died in a few months from the commencement of the treatment.

Before dismissing the subject, I would repeat my conviction that, in the management of pulmonary consumption, no remedies are so efficient as fresh air, and active, continued exercise; and that, as a general rule, all medical treatment of an energetic character had better be dispensed with. There can be no doubt that the physician may occasionally interpose palliative remedies with advantage; and symptoms often occur in the progress of the disease which require his interference; but, taking cases as they usually occur, and considering the various modes of practice which have been adopted, and are at present in use, my decided impression is, that if patients affected with phthisis were universally left to themselves, with no other medical advice than to exercise themselves freely in the open air, the general result would be a longer continuance of life, and a greater number of recoveries.

The young practitioner will often find it exceedingly difficult to carry these views into effect. The solicitude of friends, and the anxiety of the patient himself, are seldom satisfied without the constant attendance of the physician, and are always calling for the interposition of active remedies. No little firmness is requisite to resist the suggestions and solicitations of alarmed affection, and to encounter the risk of injury to his professional reputation and prospects which may result from his apparent or supposed neglect. Even the fallacious hopes which are apt to rise in his own mind, require a strong effort of judgment to be controlled. But he will find his ultimate interests to comport with a perseverance in a course of conduct compatible with his principles. If he take the proper pains to impress his views upon the minds of those concerned, though, in the temporary excitement of suffering and alarm, they may do him injustice, they will generally, in the end, when they have discovered their error, repay him for their temporary alienation by increased confidence. In the number of those whose families I am in the habit of attending, there are none who exhibit a more entire or more affectionate reliance upon my desire and ability to aid them in sickness, than some who have at a former period, in consequence of my conscientious recommendations in consumptive cases, considered me as either unskilful or negligent, and thought themselves bound to call in the aid of other counsel. In his own breast, however, the honest physician will find the surest recompense for whatever present ill will he may incur in the discharge of his duty; and this consideration should itself be sufficient to render him firm in the course which his best judgment may dictate, though opposed to the prejudices of the uninformed, and the opinions even of those whose talents and station he may greatly respect.*

* Condensed from the North American Med. and Surg. Journal.

